# Cartoon style generation of animated image scenes based on improved GAN and RetNet

**DOI:** 10.1371/journal.pone.0347014

**Published:** 2026-09-24

**Authors:** Zhenyao Jin, Lei Yang

**Affiliations:** 1 Graduate School, Cheongju University, Heongju, Korea; 2 School of Design and Art, Shandong Huayu University of Technology, Dezhou, China; Sichuan University, CHINA

## Abstract

To improve the quality and style consistency of cartoon generation in animated image scenes, this study proposes an image cartoon generation model that integrates improved generative adversarial networks and preservation networks. The model achieves collaborative optimization of local details, global structure, and diverse styles in images by introducing a multi-style mapping module, a multi-head discriminator, a Euclidean distance decay attention mechanism, and a thin plate spline geometric consistency module. This study employs two publicly available scene image datasets, setting up four typical simulation testing environments and comparing them with three mainstream image generation methods. The experiment showed that the model performed well in key indicators such as generation quality, processing efficiency, and structural preservation, with a peak signal-to-noise ratio of up to 29.2 dB, the Fréchet Inception Distance was 35.2, and a processing efficiency of 8.0 FPS. In addition, the generated cartoon image had a structural similarity index of 0.89 with the original image, and the running memory usage was controlled within 3.9GB. The image generation model has overcome the style drift and geometric distortion in existing animated cartoonization, providing a high-fidelity and low-cost automated solution for film and television production, game development, and digital content creation.

## 1. Introduction

The advancement of deep learning and computer vision has increased demand for image cartoonization in animation, digital art, games, and visual effects [1]. Cartoonization transforms real images into comic-style representations, emphasizing contours, simplifying textures, and applying artistic colors while preserving scene structure [2]. Traditional methods rely on low-level operations such as edge detection, but struggle with complex backgrounds, detail preservation, and multi-style adaptability [3]. Generative Adversarial Networks (GANs) are widely used for their realism and adaptability in image generation. For example, DGAN-MPCC: A multi-forward contrastive clustering method enhanced by dual GANs, applied to omics data. Goceri E proposed a GAN-based data augmentation method that improved classification accuracy to 93.12% on dermatoscopy images [4]. Mishra R et al. introduced an attention-based GAN with auxiliary classifier for EEG-guided image synthesis, outperforming existing methods [5]. Chen Y et al. developed a two-stage GAN for image restoration, reducing texture distortion and semantic inconsistency [6]. Xie Q et al. proposed Expo-GAN for exhibition hall style transfer, enhancing fineness and realism [7]. Gladence L M et al. created a controllable GAN framework for style transfer with adjustable intensity parameters [8]. Li L et al. designed a GAN based on global style and local high-frequency learning to retain details in complex textures. This method effectively improved the visual quality and style matching degree of the generated images in cartoonization tasks, especially outperforming existing models in detail texture restoration and edge clarity [9].

To address the local feature loss and insufficient sequence dependency modeling in image generation tasks, many researchers have begun exploring deep feature encoding and long-term dependency modeling methods. The Retentive Network (RetNet) is widely used in sequence modeling and generation tasks by efficiently capturing feature information and long-term dependencies [10]. Sun B proposed a multi-scale color correction RetNet, which integrates multi-scale color correction, information complementarity, and multi-scale feature fusion modules. This model captured local features and global information in images, improving the ability to preserve and represent complex features in generation tasks [11]. Wang B et al. applied RetNet to denoise EEG signals to address the their susceptibility to noise interference. The signal embedding strategy converted 1D EEG signals into 2D representations suitable for network processing, effectively improving the modeling ability and denoising performance of temporal features [12]. To address the high computational cost of Transformer, which limits its application in image denoising, and to creatively decouple the exponential operation bottleneck of softmax through Taylor expansion, Y. Qiu et al. dynamically compensated for the approximation error through a multi-scale attention refinement module. The computational burden was significantly reduced compared to other methods [13]. Li C et al. proposed a Transformer variant based on RetNet in the field of vision. The Gaussian mixture masks were introduced to enhance the local modeling ability on the self-attention matrix. Experiments on several small image datasets demonstrated that the proposed method effectively improved the performance of Vision Transformer, and almost did not increase additional parameters or computational costs [14].

Generative artificial intelligence is one of the most transformative technological paradigms in the current field of artificial intelligence. Its core lies in automatically generating and semantically reconstructing multi-modal content (such as text, images, audio, and video) through large-scale data-driven and probabilistic modeling. This is in line with the proposed cartoonization generation technology, both of which are based on the underlying logic of multi-modal understanding and cross-domain collaboration. To address the disconnection between technological innovation and responsibility framework in current generative artificial intelligence, Y. Sha et al. proposed a new governance framework and technological innovation path. This framework emphasized embedding traceability, explainability, and auditability designs in all stages of technology development, deployment, and use. This method could significantly enhance the traceability and decision transparency of system behavior [15]. H. Zhang et al. sought a method that combined standardization and personalization, and explored the application of generative artificial intelligence in course resources and teaching models. They applied generative artificial intelligence technology to the effective interaction between teachers and students, establishing a dynamic feedback and real-time response teaching loop. This technology could provide solid support for multi-level and personalized teaching [16].

In summary, the existing generation models have continuously evolved in terms of structural design and task adaptability, gradually breaking through the multiple constraints of traditional architectures on local details, long-range dependencies, and computational efficiency. However, there are still challenges such as insufficient collaborative modeling of local features and global semantics, insufficient robustness of cross-scale information fusion mechanisms, and limited lightweight adaptation capabilities for different tasks. To address these challenges, an animation image scene cartoonization style generation model based on GAN and RetNet is constructed. This model innovatively couples the discriminative guiding ability of the GAN and the long-range dependency modeling advantage of RetNet to solve the core bottlenecks in current research on animation image scene cartoonization, such as local texture distortion, inconsistent style transfer, and weak dynamic temporal modeling. The style-aware feature alignment module and cross-frame time RetNet encoder have been introduced to optimize semantic structure, stroke rhythm, and motion continuity. The study introduces the Thin Plate Spline Geometric Consistency (TPS-GC) module to ensure the structural rationality of the generated images through explicit geometric constraints.

The technical architecture of the research method is to model the inter-frame long-term dependency relationships through RetNet, and to achieve cross-frame geometric structure consistency constraints by combining the TPS-GC module, effectively improving the temporal coherence and deformation rationality in dynamic scenes, and ensuring that the action during style transfer is smooth and natural, and local details are stably retained. The innovations in theory, technology, and application of the research are as follows: (1) At the theoretical level, a geometric-semantic joint representation framework for cartoon-style style transfer is proposed, breaking through the traditional paradigm that only relies on pixel-level or feature-level alignment, incorporating geometric deformation modeling into the differentiable optimization loop of style transfer, and achieving unified modeling of structural consistency and style expressiveness. (2) At the technical level, a RetNet-TPS joint optimization architecture that supports multi-scale spatio-temporal alignment is constructed. Through the joint backpropagation of the learnable thin plate spline parameterized deformation field and the RetNet latent state, end-to-end geometric constraints are achieved for inter-frame motion modeling. (3) At the application level, for the animation industry, the engineering implementation of single-frame cartoonization in seconds and coherent generation of hundreds of frames is realized.

The research aims to address the core bottlenecks in current cartoonized animation image scenes, such as local texture distortion, inconsistent style transfer, and weak dynamic temporal modeling. Especially in complex motion sequences, it is prone to frame-to-frame jitter, structural collapse, and stroke breakage. Meanwhile, existing methods struggle to balance lightweight deployment and multi-style generalization capabilities, restricting their feasibility in real-time rendering and cross-platform applications.

The main challenges of this research work are that this project is oriented towards the real-time rendering requirements of the animation industrial sector, and needs to achieve Pareto optimality among single-frame fidelity, cross-frame temporal coherence, and end-side inference efficiency. Specifically, the model is required to achieve an end-to-end inference speed of ≥ 24 fps at 1080p resolution, while ensuring PSNR ≥ 28.5 dB, SSIM ≥ 0.92, LPIPS ≤ 0.18, and the model parameter size is controlled within ≤ 12M. Regarding the main challenges, the contributions of the proposed method are as follows: (1) By introducing a hierarchical feature distillation mechanism and a dynamic sparse activation strategy, the computational redundancy is reduced while ensuring semantic integrity. (2) A lightweight temporal attention module based on RetNet is designed, which reduces the temporal computational complexity while maintaining the modeling ability of long-term dependencies. (3) Integrating the operator fusion strategy based on hardware perception compresses the inference latency at the edge.

## 2. Materials and methods

In response to the missing local details, inconsistent styles, and blurry structures in the current cartoon generation task of animated image scenes, this study constructs a generation model that integrates improved GAN and RetNet. This model combines multi-style mapping, multi-head discriminator, and TPS-GC module to enhance local features and maintain global structure in two dimensions, improving the style consistency, detail fidelity, and structural integrity of image generation. The model achieves high-quality cartoon image generation in different animation scenes by optimizing multiple loss functions and modular feature fusion while maintaining image generativity.

### 2.1. Improved gan construction integrating multi-style mapping and multi-head discriminator

**Fig 1:**
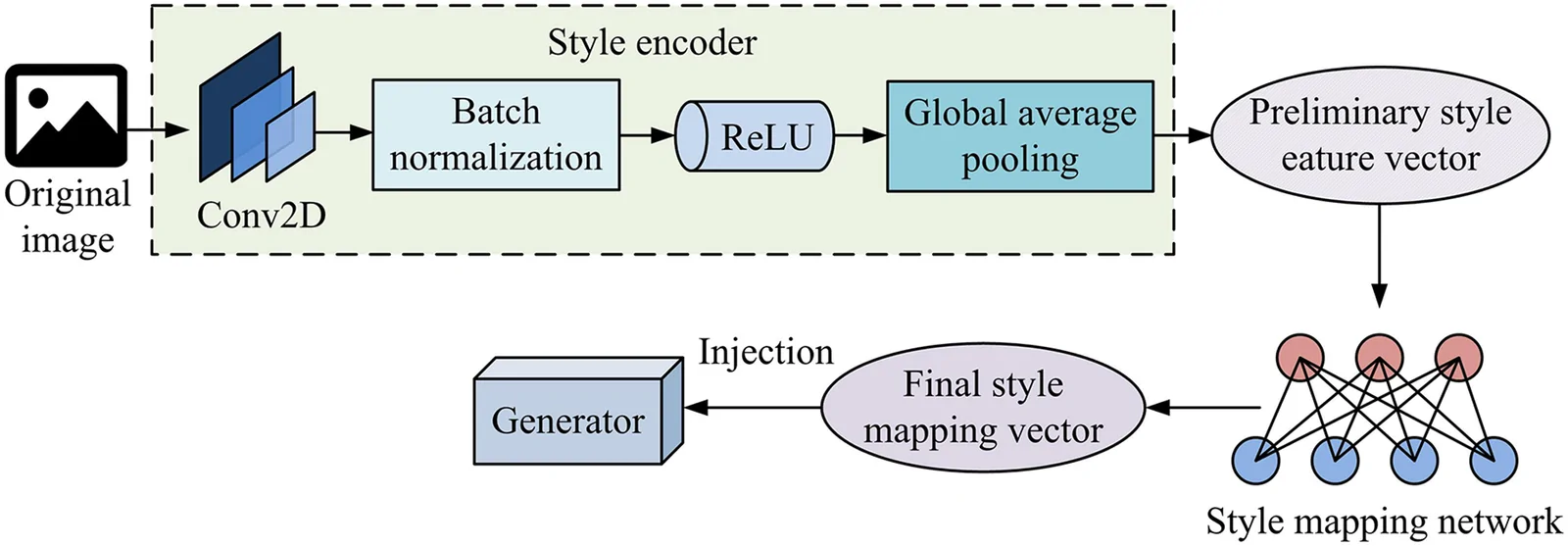
Multi-style mapping module architecture.

In the task of cartoonizing animated image scenes, traditional generation models still have shortcomings in local feature capture, texture preservation, and multi-style consistency, which can easily lead to challenges such as missing details, blurred contours, and style deviation in the generated images. To enhance the ability to express local features and global styles, this study introduces a multi-style mapping module in the generator to extract global and local style information from the input image. The overall structure is shown in Fig 1.

In Fig 1, the main function of the multi-style mapping module is to encode style features and perform diversified mapping processing on the input image, to support the generator’s flexible switching between different cartoon styles. Specifically, the module first extracts the global feature vector of the target style through a style encoder, and then generates a style condition vector by combining the content information of the input image. Subsequently, the multi-layer mapping network converts the conditional vector into a style latent representation suitable for the generator, ultimately providing diverse style guidance for subsequent image generation. The style encoder is composed of the ResNet-50 backbone network, which is initialized with pre-trained weights to enhance the model’s convergence speed and generalization ability [17]. The multi-layer mapping network adopts a residual connection structure, with each layer including normalization and nonlinear activation functions. The input layer dimension is 2,048, the output layer dimension is 512, and the hidden layer dimensions are 1024, 768, and 512 in sequence. The activation function of the hidden layers is GELU, the normalization method is LayerNorm, and the residual connection weights are initialized to 0.1. A content loss function is introduced to constrain the consistency of generated images in overall structure and semantics, as shown in equation (1).


$$\begin{array}{c} J_{content}=\alpha_{c}\cdot {\|{𝐗}_{𝐠𝐞𝐧}-{𝐗}_{𝐭𝐚𝐫𝐠𝐞𝐭}\|}_{\text{2}}^{\text{2}} \end{array}$$ (1)

In equation (1), $J_{content}$ represents the content loss. $\alpha_{c}$ is the weight of the content loss. ${𝐗}_{𝐠𝐞𝐧}$ is the generated image feature. ${𝐗}_{𝐭𝐚𝐫𝐠𝐞𝐭}$ is the target image feature. To match generated images in style representation, this study further introduces a style loss function [18], as shown in equation (2).


$$\begin{array}{c} J_{style}=\beta_{s}\cdot {\|G\left({𝐗}_{𝐠𝐞𝐧}\right)-G\left({𝐗}_{𝐭𝐚𝐫𝐠𝐞𝐭}\right)\|}_{F}^{\text{2}} \end{array}$$ (2)

**Fig 2:**
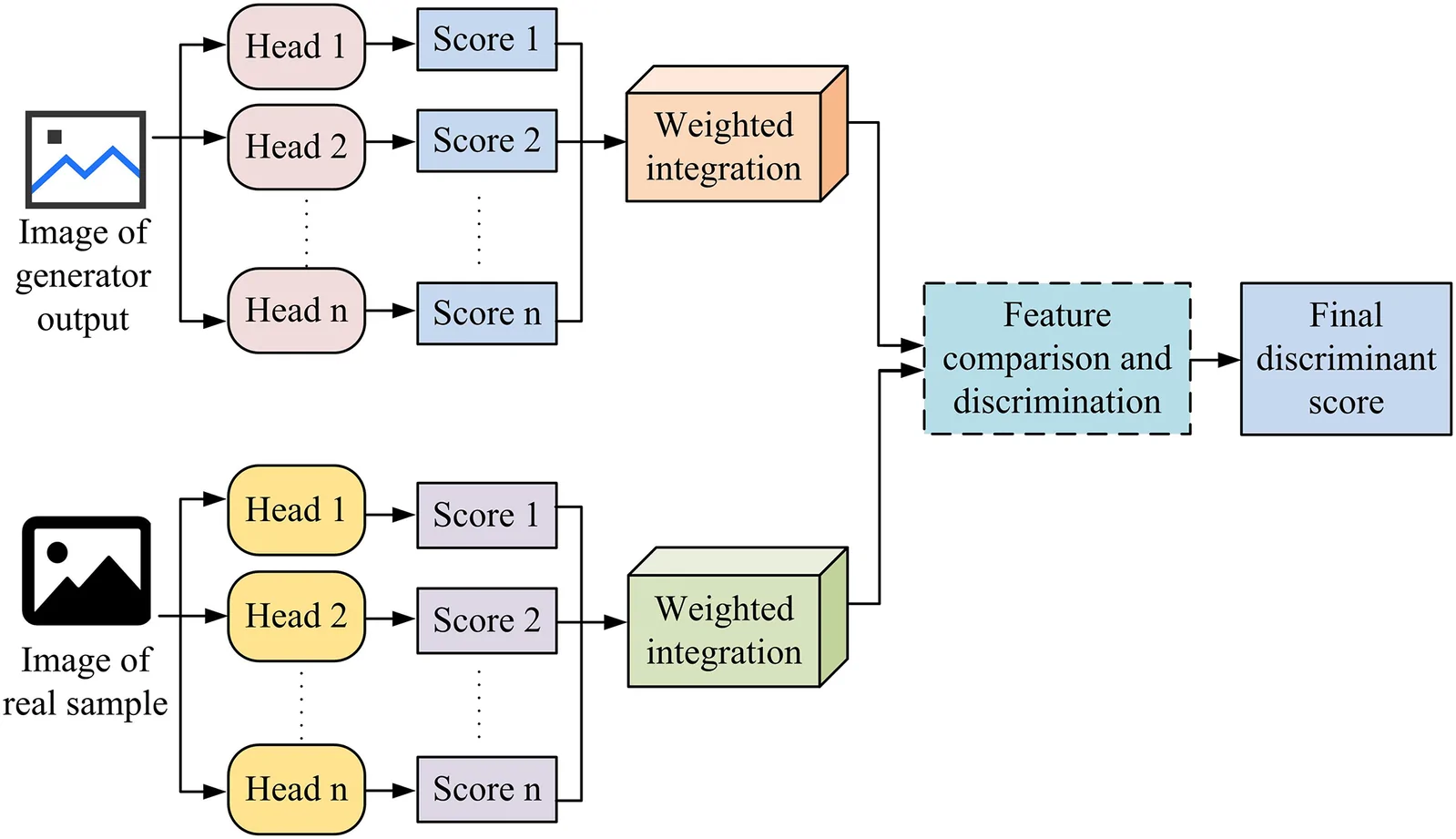
Multi-head discriminator module structure.

In equation (2), $J_{style}$ is the style loss. $\beta_{s}$ is the weight of style loss. G(·) is the style feature representation calculated through the Gram matrix. ${\|\cdot \|}_{F}$ is the Frobenius norm. The multi-style mapping module can finely model multi-level style features such as color, brushstrokes and textures by separating style features of different scales, thereby enhancing the visual consistency and artistic expressiveness of the generated images in complex animation scenes. Therefore, the research further combines the multi-head discriminator structure, enabling each discriminator head to focus on the discrimination of specific regional or style features, enhancing the model’s ability to judge the authenticity of local details and the overall style coordination. The structure of the multi-head discriminator module is shown in Fig 2.

In Fig 2, the multi-head discriminator module consists of multiple independent discriminators. Each discriminator performs discrimination on different feature sub-spaces of the image, effectively capturing local details and global structural information. In the input stage, the images output by the generator will be assigned to various discriminators for independent evaluation. The discrimination results of each discriminator are then weighted and fused to generate the final discrimination score. The multi-head scores output by the discriminator are weighted and integrated through a fusion strategy to provide more refined adversarial feedback to the generator. The multi-head discriminator module introduces a total of 1.276 million trainable parameters, accounting for 8.3% of the overall model’s parameters. It significantly improves the discrimination accuracy while maintaining high computational efficiency. Its impact on the final output lies in enhancing the inter-frame consistency of the generated images in the animation sequence, making the character actions more natural, the background texture changes more coherent, and the light effects more in line with physical laws. The adversarial loss expression of the generator is shown in equation (3) [19].


$$\begin{array}{c} J_{adv}=\gamma_{a}\cdot \left[E_{yp_{real}}\left(D\left(y\right)\right)-E_{zp_{zr}}\left(D\left(G\left(z\right)\right)\right)\right] \end{array}$$ (3)

In equation (3), $J_{adv}$ represents the adversarial loss. $\gamma_{a}$ represents the adversarial loss weight. E represents the expectation. $yP_{real}$ represents the true image distribution. $zP_{z}$ represents the input noise distribution. D(·) represents the discriminator output score. G(z) is the cartoon style feature. y is the target image. This adversarial loss guides the generator to generate more realistic images while suppressing artifacts and abnormal textures. To further preserve the contour lines and edge information of the image without loss, edge preservation loss is introduced, as shown in equation (4) [20].


$$\begin{array}{c} J_{edge}=\lambda_{e}\cdot {\|\varepsilon \left(I_{gen}\right)-\varepsilon \left(I_{target}\right)\|}_{\text{2}}^{\text{2}} \end{array}$$ (4)

In equation (4), $J_{edge}$ is the edge preservation loss. $\lambda_{e}$ is the edge preserving loss weight. ε(·) is the edge extraction operator. $I_{gen}$ and $I_{target}$ are generated images and target images. This loss ensures that the generated image maintains consistency in style while maintaining clear and distinguishable contours and structures. Under the comprehensive constraints of content loss, style loss, adversarial loss, and edge preservation loss, the definition of the total loss function is shown in equation (5).


$$\begin{array}{c} J_{total}=J_{content}+J_{style}+J_{adv}+J_{edge} \end{array}$$ (5)

In equation (5), $J_{total}$ is the total loss function. Each loss is balanced by corresponding weights to generate the structure, style, adversarial nature, and edge quality of the image. The total loss provides a complete optimization objective for the gradient update of the generator. The updated generator parameter is shown in equation (6).


$$\begin{array}{c} \theta_{G}\leftarrow \theta_{G}-\mu_{G}\cdot \nabla_{\theta_{G}}J_{total} \end{array}$$ (6)

**Fig 3:**
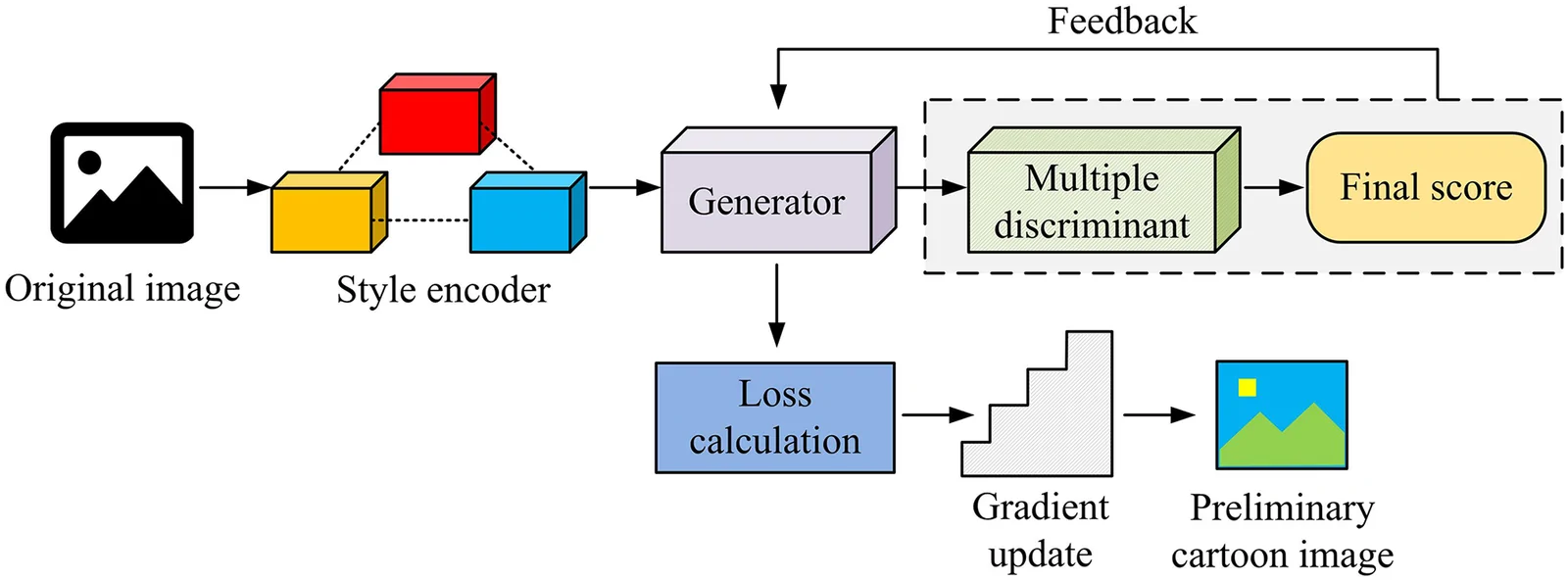
Overall architecture flow of the improved GAN.

In equation (6), $\theta_{G}$ is the generator parameter. $\mu_{G}$ is the learning rate. $\nabla_{\theta_{G}}J_{total}$ is the gradient of the loss with respect to the generator parameters. This equation is used to guide the generator to continuously optimize the quality of the generated images during the training process. In summary, the collaboration of multiple style mapping modules, multi-head discriminators, and four types of loss functions improves GAN, enhancing the local detail quality of generated images while maintaining consistency in structure, texture, and style. The overall architecture process of the improved GAN is shown in Fig 3.

In Fig 3, the GAN integrates multi-style mapping, multi-head discriminators, and generator optimization into a progressive image generation framework. It extracts style features through multi-style mapping, fuses them with content features for a multidimensional representation. The generator then produces cartoonized images, evaluated by the multi-head discriminator for style consistency and detail preservation. Content, style, adversarial, and edge preservation losses are jointly optimized, with the total loss guiding generator updates to balance structure and style. This modular design and loss optimization improve the quality and stability of cartoon animation scenes.

### 2.2. Cartoon generation model for animated image scenes integrating retnet

The enhanced GAN model improves local detail representation and style consistency in images. To better capture temporal features across frames and preserve global structure, this study incorporates a RetNet feature extraction module for long-range dependency modeling. RetNet is chosen for its strong context retention with long sequences and higher computational efficiency compared to traditional Transformers. By combining recursive and gating mechanisms, RetNet captures semantic relationships between frames in animation, improving temporal coherence. Integrating RetNet into the generator’s deep feature extraction stage strengthens the modeling of complex structures and dynamic textures while reducing flickering and distortion in multi-frame generation.

**Fig 4:**
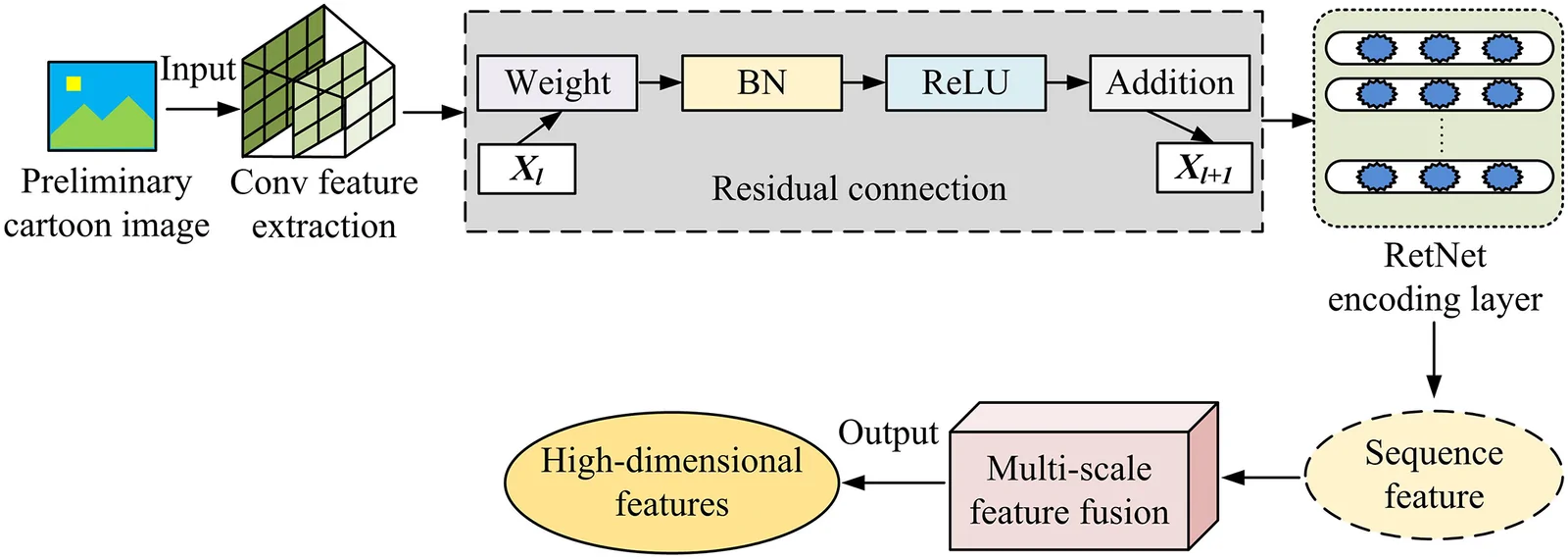
RetNet feature extraction module structure.

The attenuation coefficient is dynamically set based on the gradient stability and convergence speed at the beginning of training. The initial value is 0.85 and decreases exponentially with the number of training rounds. The Euclidean distance and semantic similarity are fused using a weighted harmonic method, with a weight ratio of 3:7 to balance the geometric structure constraints and high-level semantic consistency. This mechanism suppresses the invalid responses of distant features through a distance-sensitive gating function. The process of the RetNet feature extraction module are shown in Fig 4.

In Fig 4, The RetNet module process consists of six steps. First, it receives a cartoonized image from an improved GAN generator and standardizes its size. Second, convolutional layers extract local texture and edge features. Third, residual connections fuse low and high-level features. Fourth, the RetNet encoder serializes features to capture long-term dependencies. Fifth, a scale feature fusion module concatenates and weights multi-level features. Finally, to ensure temporal continuity, RetNet encodes frame sequences into time series, using a recursive state transfer to integrate previous frame information. Time and position encoding enhance sequence perception. A gating mechanism controls information flow, retaining key action features. The fused features are up-sampled and skip-connected, outputting a coherent cartoon animation sequence. RetNet’s temporal features are projected to match GAN generator features, reshaped spatially, and fused via weighted gated fusion with dynamic attention weights. The process is fully differentiable and optimized end-to-end. This study further defines the encoder feature mapping equation, which is used to map the convolved and serialized image features into high-dimensional representation vectors, as shown in equation (7).


$$\begin{array}{c} {𝐇}_{𝐫}=f_{RetNet}(I;\theta_{r}) \end{array}$$ (7)

In equation (7), ${𝐇}_{𝐫}$ is the multi-scale feature output by the RetNet encoder. $f_{RetNet}$ is the RetNet feature extraction function. I is the input image. $\theta_{r}$ is a parameter of the RetNet module. Based on this feature, the self-attention mechanism with Euclidean distance decay is shown in equation (8) [21,22].


$$\begin{array}{c} {𝐀}_{𝐝}=Softmax(\frac{{𝐇}_{𝐫}{{𝐇}_{𝐫}}^{⊤}}{\sqrt{d}}-\delta_{a}𝐃) \end{array}$$ (8)

**Fig 5:**
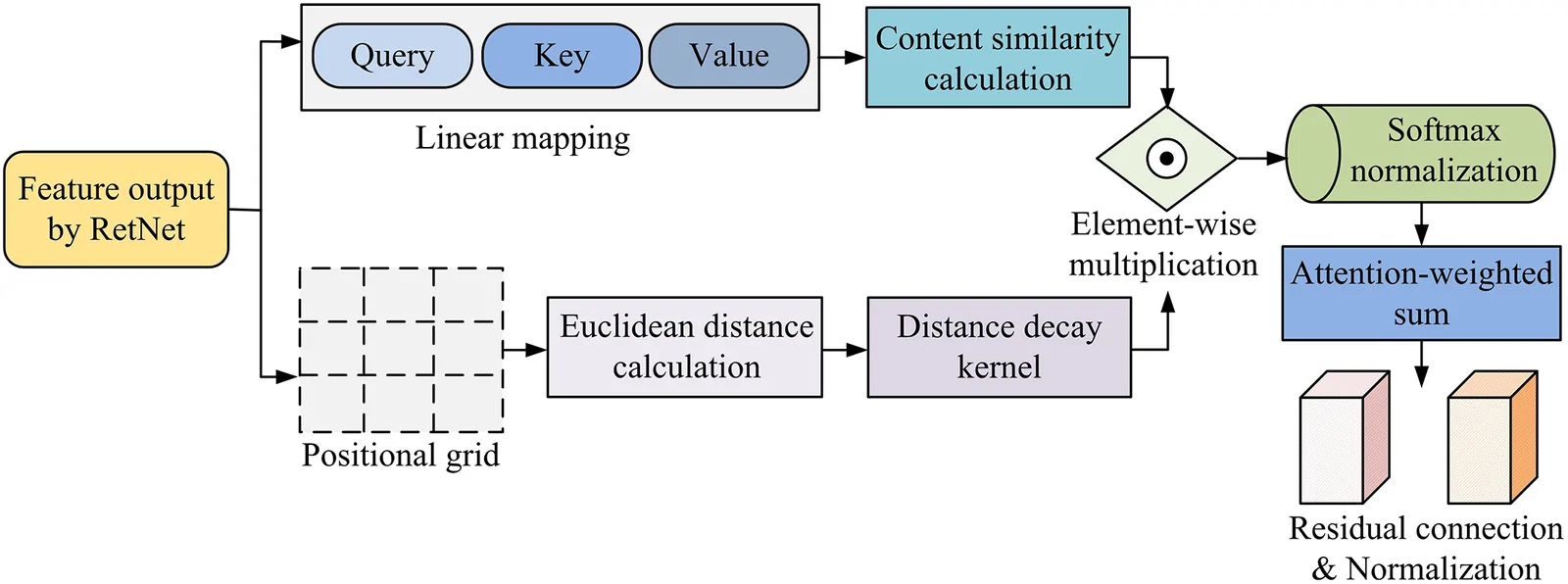
Euclidean distance-decayed self-attention module flow.

In equation (8),${𝐀}_{𝐝}$ is the attenuated self-attention matrix. Softmax(·) is to convert the similarity matrix into a probability distribution. ${𝐇}_{𝐫}{{𝐇}_{𝐫}}^{⊤}$ is the similarity between features. d is the feature dimension normalization factor. $\delta_{a}$ is the self-attention decay coefficient. 𝐃 is the Euclidean distance matrix between pixels. This self-attention mechanism can suppress long-distance interference while retaining remote dependencies, improving the consistency between local features and global structure in animated scenes [23,24]. Compared with the self-attention mechanism, this mechanism combines Euclidean decay, significantly enhancing the perceptual accuracy of spatial continuity between animation frames while retaining global modeling capability. The motivation for choosing this mechanism is that it can effectively model pixel level spatial continuity constraints between animation frames, especially in scenes with rapid motion or significant local deformation. Compared with traditional self-attention mechanisms, it can reduce the structural misalignment rate. The structure of the self-attention module is shown in Fig 5.

In Fig 5, this model first receives the feature output of RetNet, and then performs a linear projection to obtain the Q, K, and V vectors: the content similarity branch calculates the semantic similarity matrix, and the distance attenuation branch calculates the Euclidean distance matrix and assigns weights. After fusion, a comprehensive similarity matrix is generated, and the distance-aware attention weights are obtained through Softmax, and the weighted aggregated values are used to obtain an enhanced context representation. Finally, it is sent to the time consistency modeling module through residual connection and normalization. In addition, the study introduces the TPS-GC module to constrain the smoothness and rationality of the geometric structure during the cartoonization process, avoiding deformation. This module simulates the deformation of key points through thin plate spline interpolation, combines graph convolution, jointly optimizes node displacements and edge weights, introduces bending energy regularization to suppress distortion, and finally outputs coordinated features for the decoder to reconstruct high-quality cartoon images. It can collaboratively optimize semantic representation and geometric structure, while preserving the original content features and maintaining the rationality of the key topological structure. The definition is shown in equation (9).


$$\begin{array}{c} {𝐓}_{𝐩}=TPS({𝐇}_{𝐝};n_{p}) \end{array}$$ (9)

**Fig 6:**
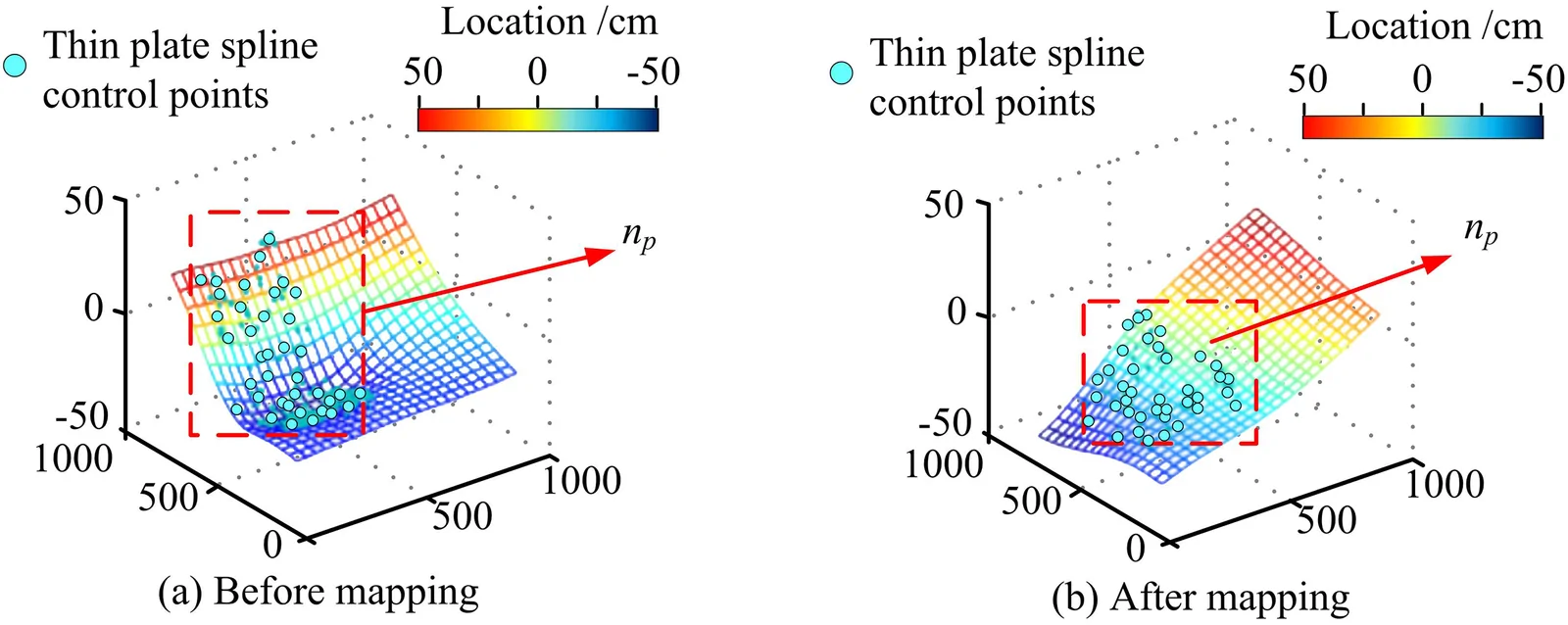
TPS-GG module structural principle.

In equation (9), $n_{p}$ represents the mapping matrix output by the TPS-GG module. ${𝐇}_{𝐝}$ represents the feature representation after self-attention processing. $n_{p}$ represents the number of control points of the thin plate spline. TSP(·) is the key point map used for TSP construction. The specific principle of this module is shown in Fig 6.

In Fig 6, the TPS-GG module performs local geometric mapping and adjustment on the self-attention processed features to enhance the structural integrity and style adaptation of the generated images. In Fig 6(a), the module first generates $n_{p}$ control points on the feature map. In Fig 6 (b), the local deformation matrix is calculated, and then the final output mapping and adjusted feature matrix are formed through weighted fusion, providing reliable geometric guidance for subsequent image generation. This module is easy and efficient to operate, balancing structural fidelity and style adaptation. After the TPS-GC module outputs features, this study integrates them with the style vectors of the multi-style mapping module. The fusion is shown in equation (10) [25].


$$\begin{array}{c} {𝐅}_{𝐜𝐬}=\lambda_{c}{𝐇}_{𝐫}+\lambda_{s}{𝐅}_{𝐬} \end{array}$$ (10)

In equation (10), ${𝐅}_{𝐜𝐬}$ is the fused feature. ${𝐅}_{𝐬}$ is the output of the multi-style mapping module. $\lambda_{c}$ and $\lambda_{s}$ are both fusion weights. To further constrain the geometric consistency of generated images, the TPS-GG consistency loss is introduced, as shown in equation (11) [26].


$$\begin{array}{c} J_{TPS}=\lambda_{t}{\|{𝐓}_{𝐩}-{𝐅}_{𝐜𝐬}\|}^{\text{2}} \end{array}$$ (11)

In equation (11),$J_{TPS}$ is the TPS-GG consistency loss. $\lambda_{t}$ is the loss weight. The study combines five loss functions, including style loss, adversarial loss, edge-preserving loss, and TPS-GG consistency loss, to derive the final loss function. This loss function is applied during the training of the overall model. The Adam optimizer is used, with an initial learning rate of 2 × 10^−4^. After 50 epochs, a cosine annealing schedule with a decay rate of 0.98 is implemented. The batch size is set to 16, and training proceeds for 100 epochs. All experiments are conducted on an NVIDIA A100 GPU cluster, with approximately 18.2GB of GPU memory usage per card. The weight selection of each loss function adopts a combination of grid search and ablation experiments to evaluate the impact of different weight combinations on the PSNR, LPIPS, and human ratings of the validation set. This module contains 32 embedded learning style vectors, each with a dimension of 512, and the total number of parameters is approximately 16,400. The influence of this module in the framework is to significantly enhance the model’s generalization ability for diverse artistic styles, especially in handling unseen calligraphy styles, where it improves the transfer accuracy rate.

The final weights are determined as follows: style loss = 0.8, adversarial loss = 1.2, edge-preserving loss = 0.6, TPS-GG consistency loss = 1.0, and the remaining term = 0.4. Integrating these elements, the overall loss function is shown in equation (12).


$$\begin{array}{c} J_{fusion}=0.4\cdot J_{content}+0.8\cdot J_{style}+1.2\cdot J_{adv}+0.6\cdot J_{edge}+1.0\cdot J_{TPS} \end{array}$$ (12)

**Fig 7:**
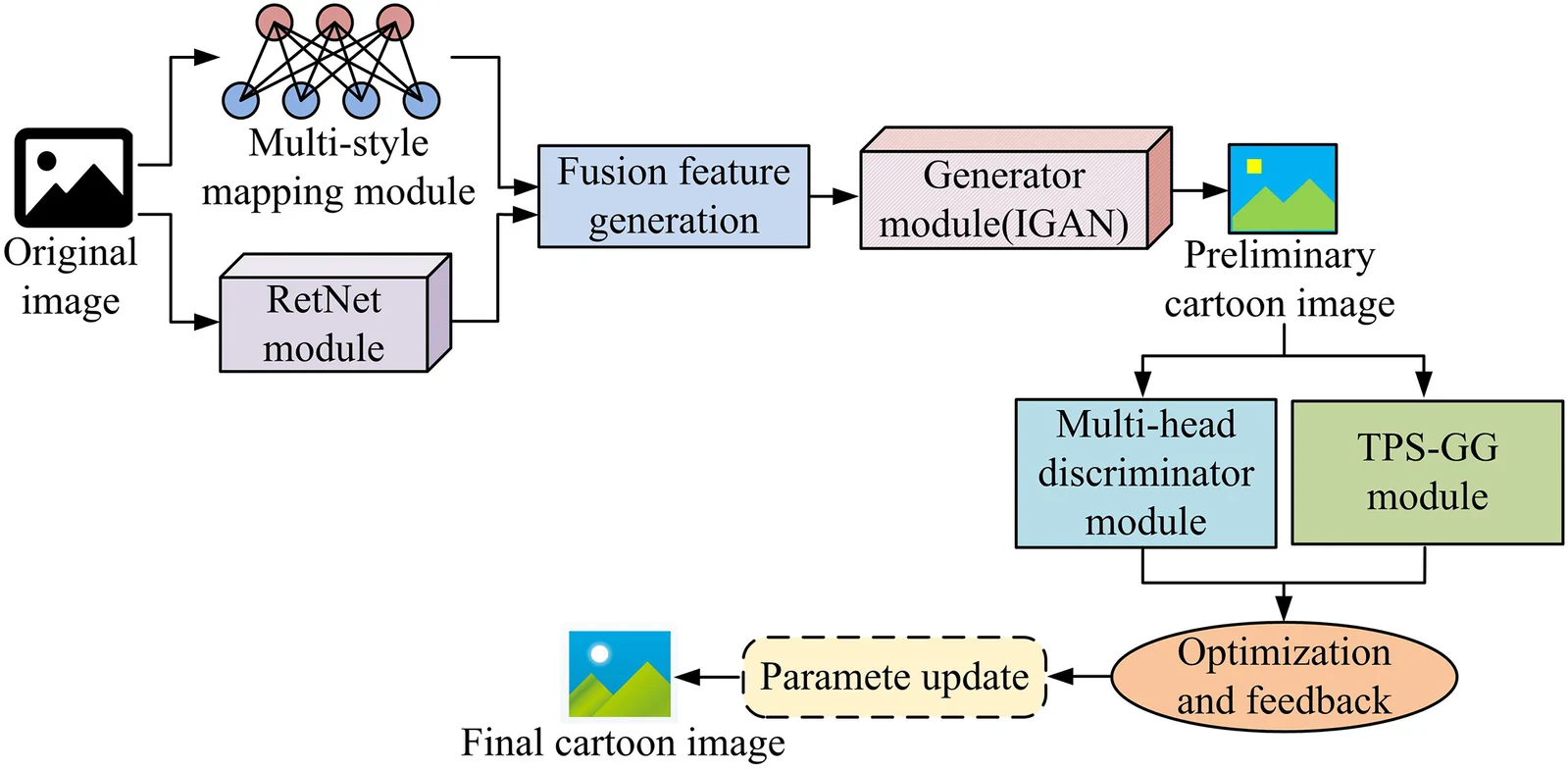
Overall architecture of the IGRAC model.

In equation (12), ${\text{J}}_{\text{fusion}}$ represents the total loss function. The training parameters of the model are selected through grid search and ablation experiments to ensure the convergence stability and generalization ability of each loss term on the validation set. The overall structure of the Improved GAN and RetNet-based Animated Scene Cartoonization Generation Model (IGRAC) based on the total loss of the fusion network is shown in Fig 7.

In Fig 7, the model combines an improved GAN module with a feature extraction and fusion mechanism based on RetNet for cartoonization generation of animated image scenes. The process first sends the input image to the multi-style mapping module to extract and encode global and local style features. Subsequently, these style features are fused with the content features output by the RetNet encoder to form a comprehensive feature map. The fusion feature input generator generates preliminary cartoonized images and evaluates local and global consistency through multi-head discriminators. Meanwhile, the TPS-GG module performs geometric mapping on the fused features to enhance the consistency of style transformation. Generator optimization relies on the combined effect of content loss, style loss, adversarial loss, edge preservation loss, and TPS-GG consistency loss. After multiple iterations, the model parameters are updated to continuously improve the generation effect. This modular and layered design enables the IGRAC model to achieve high-quality cartoon style transformation while maintaining image structural integrity, while also considering texture details and style consistency, making it suitable for generating tasks in different animation scenes. The following are the relevant discussion contents about potential target conflicts that may occur during the collaborative optimization process of multiple losses: When the weight of the style loss is too high, it is prone to cause texture distortion and edge blurring. An overly strong adversarial loss leads to high-frequency noise and artifacts. To address this issue, the research introduces a dynamic weight adjustment mechanism. During the training process, the weight ratio is adaptively adjusted according to the gradient norm of each loss term to ensure a balance between style fidelity and structure preservation. A gradient clipping strategy is designed to limit the excessive parameter update amplitude dominated by the adversarial loss.

The study combines five modules: multi-style mapping module, multi-head discriminator module, GAN, RetNet, and TPS-GG. The multi-style mapping module is responsible for decoupling and adapting diverse cartoon styles. The multi-head discriminator module achieves hierarchical discrimination between local texture authenticity and global semantic consistency. The GAN framework provides strong prior generation and adversarial driven optimization capabilities. The RetNet encoder, with its advantage in long-range modeling, accurately captures scene structure and spatial dependencies. The TPS-GG module uses differentiable geometric calibration to accurately correct the spatial alignment relationship between style and content, alleviating the style misalignment caused by perspective distortion or posture changes.

To ensure the quality of generation while reducing inference delay, a lightweight adaptation mechanism is proposed. This mechanism adopts a dual-path compression strategy of structured pruning and knowledge distillation to simplify the main body of the RetNet encoder and generator. While retaining the key attention heads and local convolutional channels, sparse pruning is performed on the non-key channels. The joint inference capability of the RetNet encoder and generator is transferred to the lightweight student network using the teacher-student distillation framework. Additionally, a style-aware attention transfer loss is introduced during the knowledge distillation process to guide the student network to replicate the style response patterns of the teacher network at key feature layers, thereby enhancing the generation fidelity of the lightweight model in complex texture regions. During the entire model training process, the generator contains 12.7 million trainable parameters, the multi-head discriminator has a total of 1.276 million parameters, and the style mapping module accounts for 16,400; the parameters of the RetNet encoder are 8.92 million, and the TPS-GG calibration module only contains 4,300 parameters. The overall parameter quantity is controlled at 22.9167 million.

### 2.3. Ethics statement

This study does not involve any human participants, human tissue, or human data. All images used in this research were obtained from publicly available datasets and do not require informed consent. No ethical approval was required for this study.

## 3. Results and analysis

This study established a stable experimental environment and determined the optimal configuration of the IGRAC model in cartoon image generation tasks through hyperparameter selection testing of content loss weights and self-attention attenuation coefficients. Subsequently, multiple ablation tests were conducted to evaluate the impact of each module on image quality and structural fidelity. Further performance comparison tests were conducted on two datasets to evaluate the image generation quality, processing efficiency, memory usage, and other indicators in different simulation environments. This study also constructed four typical simulation environments and verified the superiority and efficiency of the IGRAC model in image generation tasks through comprehensive indicators, proving its stability and generalization ability in different environments.

### 3.1. Parameter selection and ablation testing of igrac cartoon image generation model

To validate the performance of the constructed IGRAC model, two publicly available scene image datasets were selected as test data sources: the ADE20K: MIT Scene Parsing Benchmark (ADE20K) and the Cityscapes Dataset for Semantic Urban Scene Understanding (Cityscapes). ADE20K set of official data link is: https://ade20k.csail.mit.edu/. ADE20K offers a wide range of indoor and natural scenes, while Cityscapes focuses on dynamic street scenes and fine-grained semantic segmentation labels. The two complement each other well and can comprehensively evaluate the collaborative performance of IGRAC in three aspects: structure understanding, edge preservation, and style transfer. The ADE20K dataset was released by the Computer Vision and Artificial Intelligence Laboratory at the Massachusetts Institute of Technology and contains over 27,000 images covering more than 300 different scenes. The Cityscapes dataset was released by the University of Freiburg in Germany and includes urban street scene images from 50 cities. The training/test split ratio of the ADE20K and Cityscapes datasets is 8:2, meaning 80% for training and 20% for testing. During the data preprocessing stage, all images are uniformly re-sized to a resolution of 512 × 512 and enhanced with random horizontal flipping, color jittering, and Gaussian noise to improve robustness. The cartoon style annotations were completed collaboratively by 3 senior animators to ensure compliance with three standards: simplicity of lines, coherence of color blocks, and semantic recognizability. The consistency of annotations was verified by Cohen’s Kappa test and reached 0.89, confirming the reliability of the annotation quality. The Cityscapes dataset was released by the University of Freiburg in Germany and includes street view images from 50 cities. Each set of comparative experiments was conducted with 10 independent repeated trials, and the same random seed was used in each trial to ensure reproducibility. Each set of comparative experiments was run under the same hardware configuration to avoid deviations caused by environmental differences. To ensure that the sample size was sufficient to support the statistical power, the sample size of each experiment was calculated using the G*Power 3.1 software. The effect size d was set at 0.5, the significance level α was set at 0.05, and the statistical power 1 – β was set at 0.95. The minimum required sample size was determined to be 28. The actual 10 repetitions × each round ≥ 30 test images were conducted, which fully met the statistical requirements. This study established a stable experimental environment for model training and testing, with specific configurations shown in Table 1.

**Table 1:** Experimental environment configuration for the IGRAC model.

Category	Key specifications
CPU	AMD Ryzen Threadripper 5995WX
GPU	NVIDIA RTX 4090
RAM	128GB DDR5
Storage	2TB NVMe SSD + 8TB HDD
OS	Ubuntu 22.04 LTS
DL framework	PyTorch 2.1 + CUDA 12.1
Data processing	OpenCV 4.8, Pillow 10.0, NumPy 1.27

**Fig 8:**
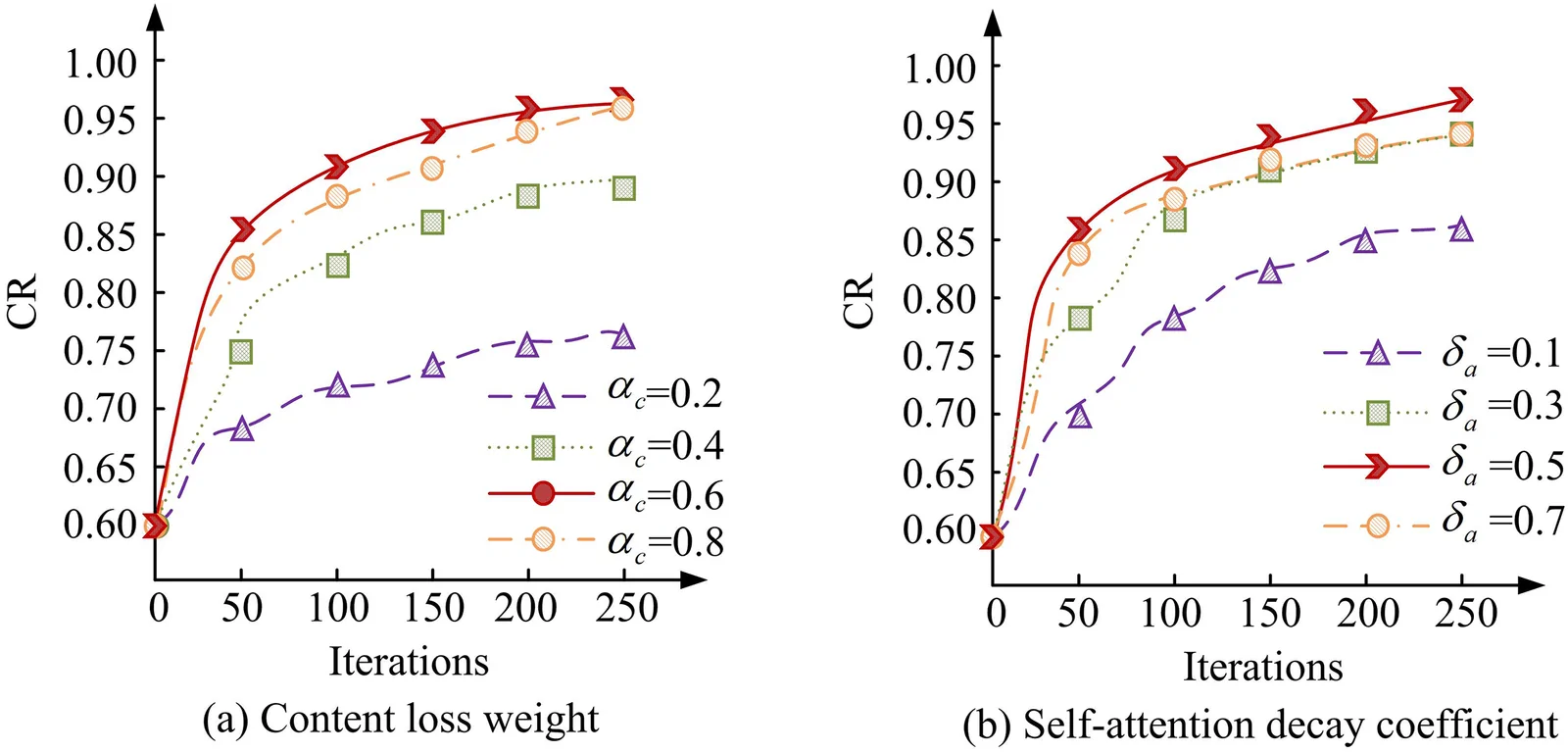
Hyperparameter selection test.

To ensure the generation quality and structural fidelity of the IGRAC model in animation scene cartoonization tasks, this study first conducted value selection tests on two key hyperparameters that affect model performance: content loss weight $\alpha_{c}$ and self-attention decay coefficient $\delta_{a}$. Taking Content Retention (CR) as an indicator, the test results are shown in Fig 8.

**Fig 9:**
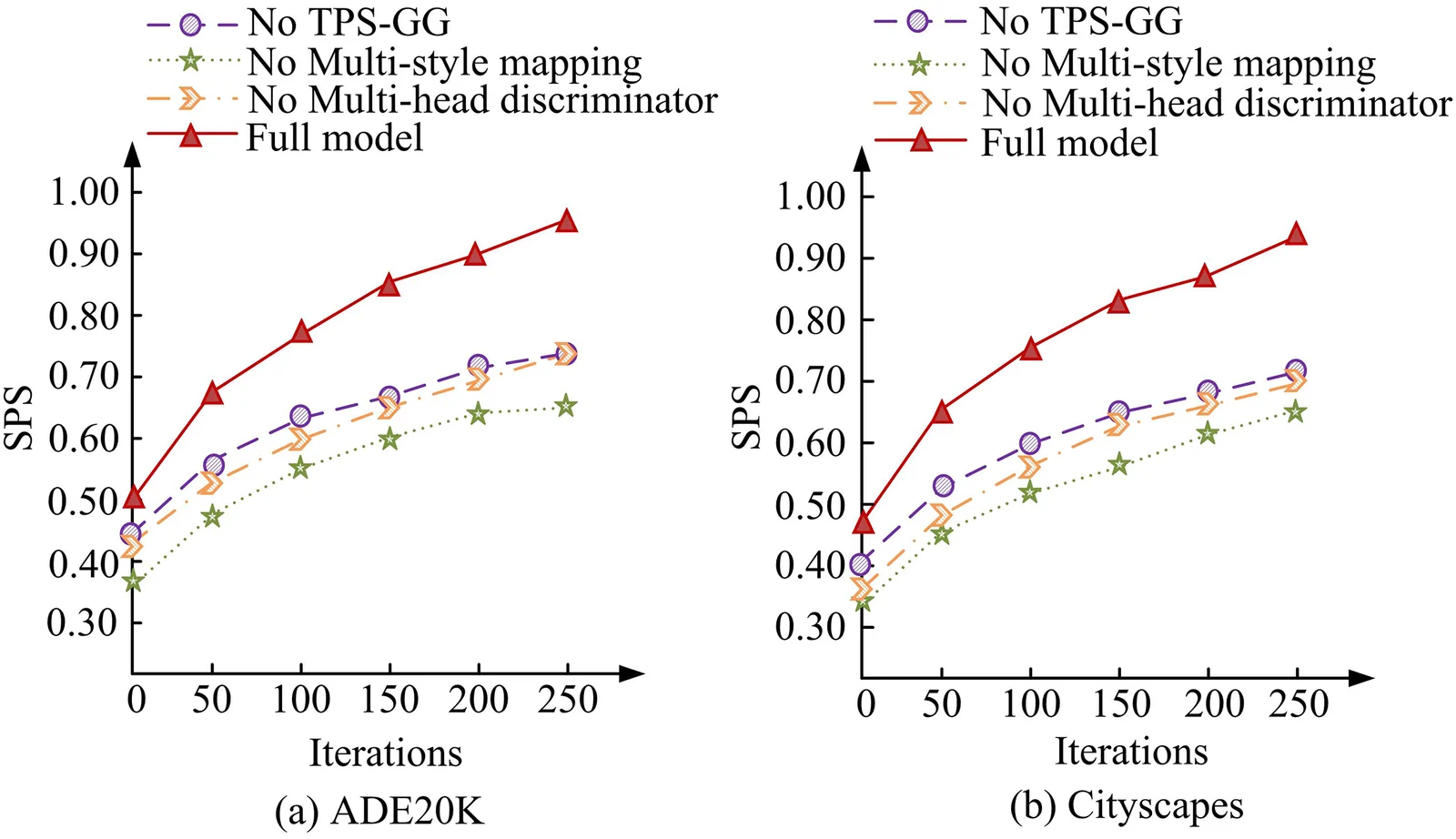
Ablation test results on two datasets.

Fig 8a shows the test results of different $\alpha_{c}$ under the premise of $\delta_{a}$ being fixed. Fig 8b shows the results of different $\delta_{a}$-coefficients. In Fig 8a, when $\alpha_{c}$=0.2, the model converges slowly, and the final CR value is only 0.76, indicating the worst performance. When $\alpha_{c}$=0.4, the model converges faster and the final CR reaches 0.89. When $\alpha_{c}$=0.6, the convergence speed is the fastest and the CR reaches 0.97, indicating the best effect. When $\alpha_{c}$ further increases to 0.8, the initial convergence is faster, but the later improvement slows down, and the final CR is 0.93. In Fig 8b, when $\delta_{a}$=0.1, the model converges slowly, and the final CR is 0.86. When $\delta_{a}$=0.3, the convergence accelerates and the final CR is 0.94. When $\delta_{a}$=0.5, the model converges the fastest and the CR reaches 0.97, indicating the best performance. In summary, this study determines to set $\alpha_{c}$ and $\delta_{a}$ to 0.6 and 0.5 as the optimal hyperparameter combinations for the IGRAC model during training, while ensuring significant performance differences between different selected values and highlighting the advantages of the optimal values. This study conducted model ablation tests on two datasets using Structural Preservation Score (SPS) as an indicator, as shown in Fig 9.

Fig 9a shows the SPS of different ablation models with training iterations on the ADE20K dataset. Fig 9b shows the changes in SPS on Cityscapes. In Fig 9(a), the model without TPS-GG module only converges to 0.73. The model that removes the multi-style mapping module has a lower initial score and converges to 0.66 after 250 iterations. The model without the multi-head discriminator module performs moderately and eventually converges to 0.73. In contrast, after 250 iterations, the IGRAC achieves an SPS of 0.95, which is higher than that of other models. Similarly, on the Cityscapes dataset in Fig 9b, the SPS variation trends of different ablation models are basically consistent with Fig 9(a). The complete IGRAC model reaches 0.94 after 250 iterations, outperforming other ablation models. Under the synergistic effect of TPS-GG, style mapping, and multi-head discriminator, the model can effectively maintain the structural and texture integrity of the animation scene. This study continues to test using Peak Signal-to-Noise Ratio (PSNR), Structural Similarity Index Measure (SSIM), and Fréchet Inception Distance (FID) as indicators, as shown in Table 2.

**Table 2:** Performance comparison of ablation models on two datasets.

Data set	Model	PSNR/dB	SSIM	FID
ADE20K	No TOS-GG	18.5	0.64	72.4
	No Multi-style Mapping	17.8	0.60	78.6
	No Multi-head discriminator	19.2	0.67	70.1
	IGRAC	24.8	0.89	35.2
Cityscapes	No TOS-GG	18.2	0.62	74.0
	No Multi-style Mapping	17.5	0.59	80.3
	No Multi-head discriminator	19.0	0.65	71.5
	IGRAC	24.5	0.88	36.0

In Table 2, IGRAC achieves the best performance in PSNR, SSIM, and FID metrics on ADE20K and Cityscapes. On the ADE20K dataset, its PSNR reaches 24.8, SSIM is 0.89, and FID decreases to 35.2, which is 7.0, 0.29, and 43.4 higher than the model without the multi-style mapping module. On the Cityscapes dataset, the complete model also maintains a leading PSNR of 24.5, SSIM of 0.88, and FID of 36.0. The multi-style mapping module has the greatest impact on overall performance. When this module is missing, PSNR and SSIM decrease significantly, while FID increases significantly. This module plays an indispensable role in style consistency and feature mapping.

### 3.2. Simulation testing of igrac cartoon image generation model

**Fig 10:**
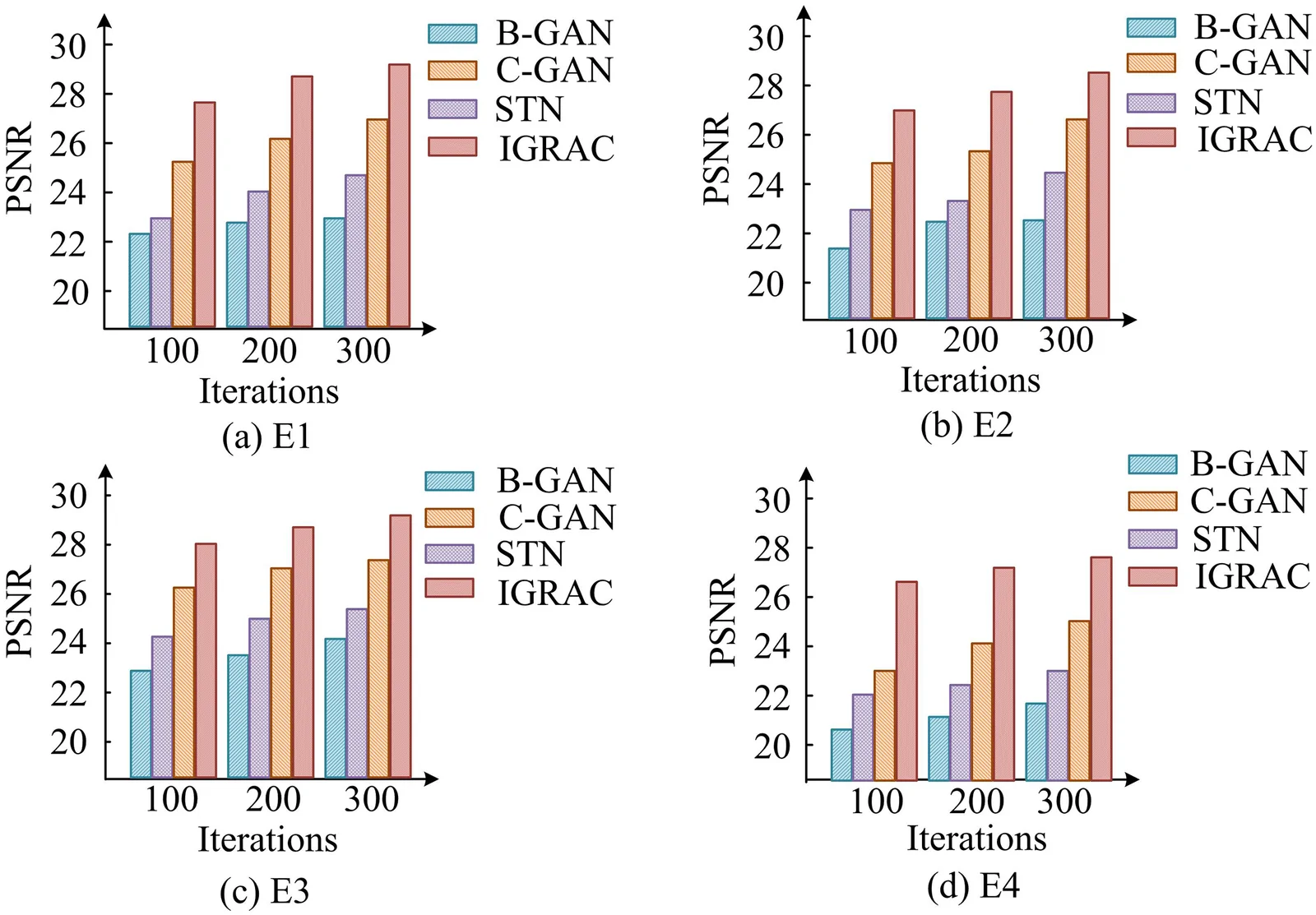
PSNR comparison of four models under four simulated environments.

To verify the performance of the IGRAC model in different simulation environments, this study simulates four typical simulation testing environments based on a dataset. E1: Standard urban street view environment; E2: Natural landscape environment; E3: Indoor scene environment; E4: Image environment containing human characters. In addition, this study selects three models suitable for image cartoonization tasks: the traditional Baseline GAN (B-GAN), the Style Transfer Network (STN), and the specially designed Cartoonization GAN (C-GAN). This study first compares the PSNR, as shown in Fig 10.

**Fig 11:**
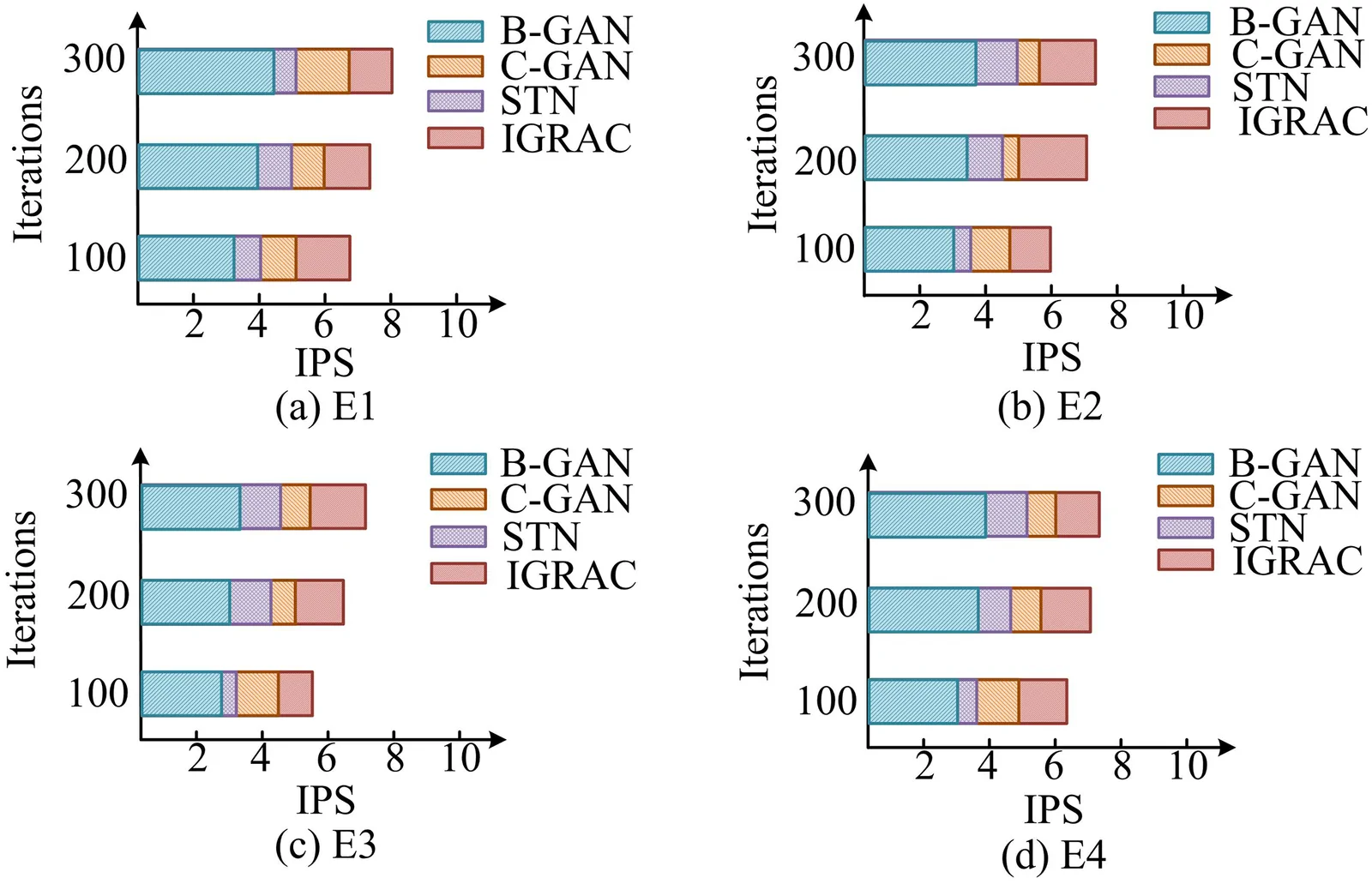
IPS comparison of four models under four simulated environments.

Figs 10a to (d) show the PSNR value with training iterations for four models in four image generation simulation environments. In Fig 10(a), in E1, the PSNR value of IGRAC is 29.0 dB, while the PSNR values of other models at 300 iterations do not exceed 27 dB. In Fig 10(b), in E2, the PSNR value of IGRAC is 28.5 dB, which is significantly better than that of C-GAN (26.8 dB), STN (25.3 dB), and B-GAN (23.0 dB). In E3 and E4, IGRAC consistently outperforms other models in all iterations, with PSNRs of 29.2 dB and 27.8 dB. The results demonstrate the high-quality output capability of the IGRAC model in different image generation tasks. This study continues to compare Images Processed Per Second (IPS), as shown in Fig 11.

**Fig 12:**
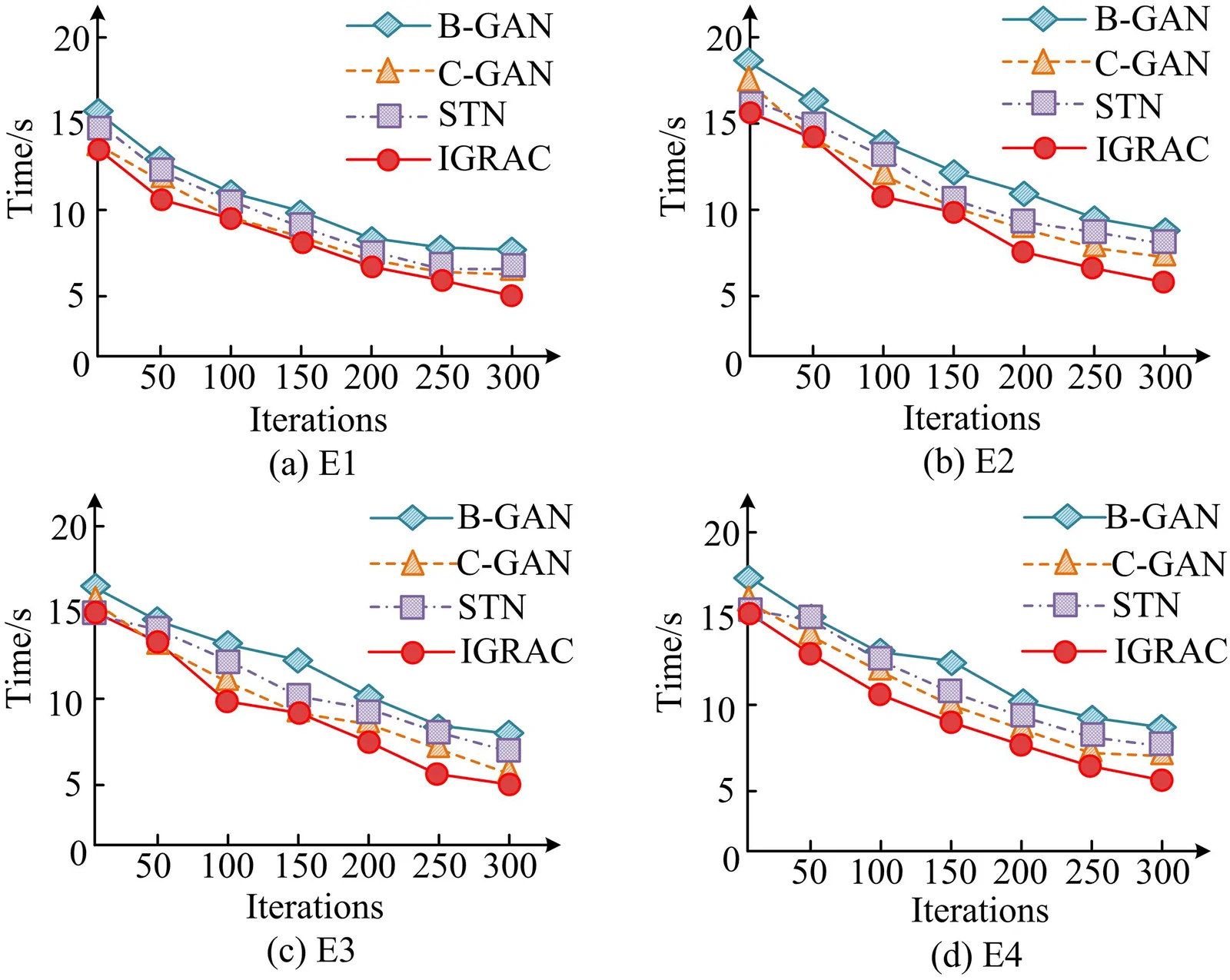
Processing time comparison of four models under four simulated environments.

Figs 11a to (d) show the IPS value of four models as they vary with training epochs. In Fig 11a, in E1, IGRAC has already reached 6.8 IPS in the 100th round and increased to 8.0 IPS in the 300th round, demonstrating strong computational processing capabilities. In contrast, the processing capability of B-GAN is relatively stable, reaching only 4.2 IPS after 300 iterations. In Figs 11b and (c), the performance of STN and C-GAN is moderate, with STN reaching a maximum of 5.5 IPS, while C-GAN stabilizes at 6.0 IPS at 300 iterations. In E4 shown in Fig 11(d), IGRAC ultimately reaches 7.5IPS. Overall, IGRAC has demonstrated the strongest processing capability in different environments, verifying its superiority and efficiency in image cartoonization tasks. This study continues to test using processing time as an indicator, as shown in Fig 12.

Figs 12a to (d) show the processing time of the model changing with training iterations. From Fig 12(a), in E1, the processing time of IGRAC is 5.7 seconds, which is lower than that of other models. Similarly, under E2, the processing time of IGRAC is 6.3 seconds, which is better than that of C-GAN (7.2 seconds). In E3 and E4, IGRAC also shows significant advantages, leading other models by approximately 1 second. IGRAC exhibits strong training efficiency in different environments, with significantly lower processing time compared to the other three models. This indicates the efficiency of IGRAC in image generation tasks, which can improve overall computational performance while ensuring image quality. This study further compares four models in memory usage, running efficiency, and the quality of generated images with the expected accuracy of the target, as shown in Table 3.

**Table 3:** Performance comparison of four models in different simulation environments.

Environment	Model	Memory usage/GB	Running efficiency/FPS	Accuracy/%
E1	B-GAN	4.2	15.2	87.5
	STN	4.1	16.5	89.2
	C-GAN	4.3	17.0	90.0
	IGRAC	3.9	18.0	92.3
E2	B-GAN	4.3	14.8	85.3
	STN	4.2	15.7	87.6
	C-GAN	4.4	16.5	89.0
	IGRAC	4.0	17.3	91.2
E3	B-GAN	4.1	13.5	83.5
	STN	4.0	14.2	85.1
	C-GAN	4.2	15.0	87.0
	IGRAC	3.8	16.2	89.8
E4	B-GAN	4.0	12.8	80.5
	STN	3.6	13.6	82.3
	C-GAN	4.1	14.2	84.5
	IGRAC	3.7	15.0	87.1

In Table 3, the IGRAC model shows the best performance in memory usage, running efficiency, and accuracy in all four environments. In E1, IGRAC has the smallest memory footprint, only 3.9GB, and has the highest running efficiency of 18.0 FPS with an accuracy of 92.3%, which is better than that of the other three models. In E2, the memory usage of IGRAC is 4.0GB, the running efficiency is 17 FPS, and the accuracy is 91.2%. In E4, IGRAC has a memory usage of 3.7GB, a running efficiency of 15.0 FPS, and an accuracy of 87.1%, still maintaining its leading position. In summary, the IGRAC model demonstrates optimal overall performance in four different simulation environments, particularly in memory usage and operational efficiency, validating its superiority in image generation tasks.

**Fig 13:**
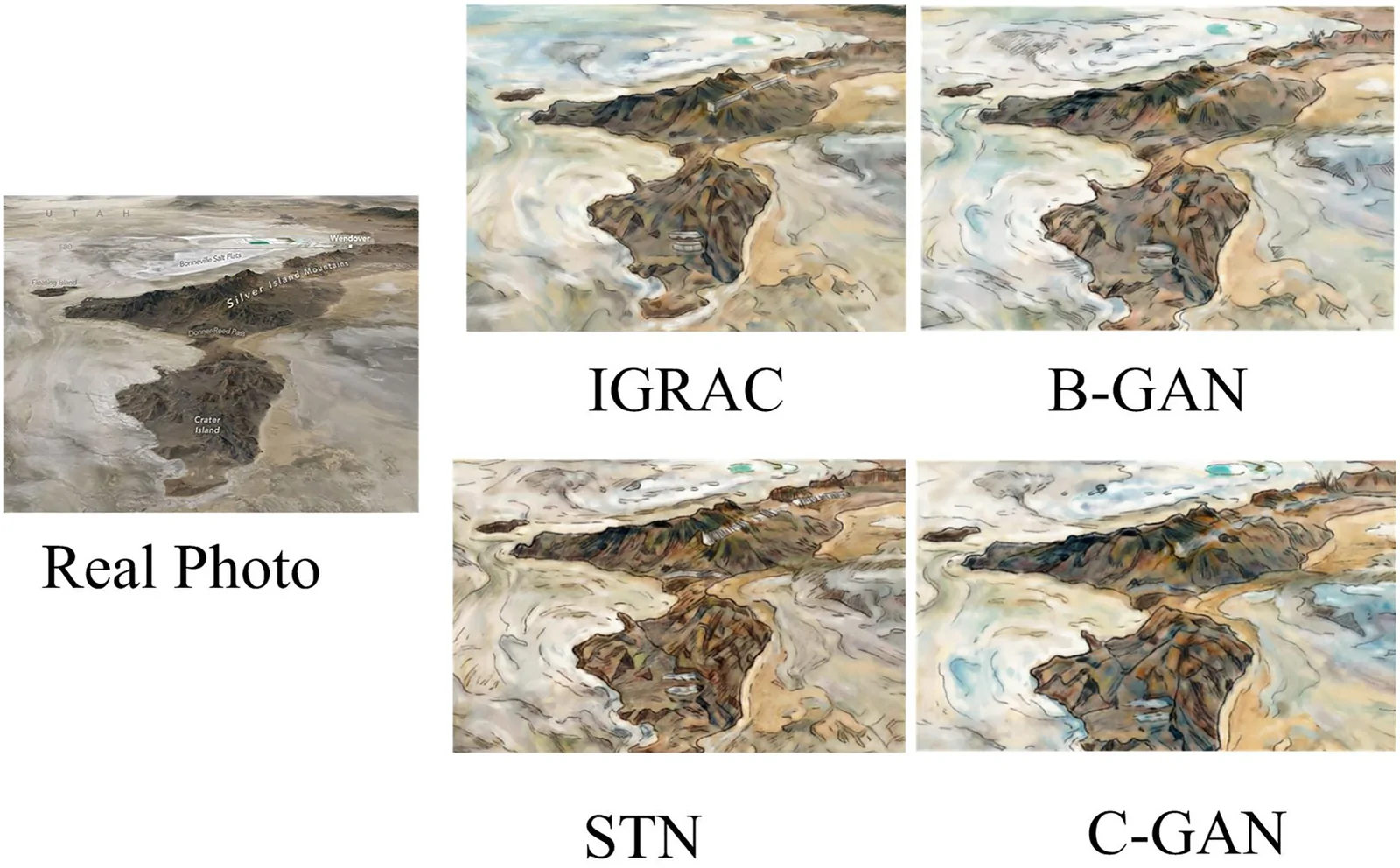
Cartoon generated visual example results for each model scene(The Photo from: https://science.nasa.gov/earth/earth-observatory/what-lake-bonneville-left-behind/).

To further visualize the performance differences of each model in different environments, the study provides visual examples, as shown in Fig 13.

As shown in Fig 13, the study compared the cartoon image generation effects of IGRAC with B-GAN, STN, and C-GAN under complex backgrounds and low-light conditions. The qualitative results indicated that IGRAC performed exceptionally well in terms of detail restoration and style consistency: its edge processing was more natural, the color matching had a more artistic feel, and it could still maintain clear texture structures under low-light conditions without obvious distortion or blurring, verifying the model’s strong feature extraction and cross-domain transfer capabilities. To quantitatively analyze the results of contour clarity, style consistency, and detail loss, and demonstrate the superiority of IGRAC in subjective visual quality, this paper uses three indicators: LPIPS, FID, and CLIP scores for evaluation. The experimental results show that in the E1-E4 environments, the average LPIPS value of IGRAC is 0.128, significantly lower than that of B-GAN (0.216), STN (0.193), and C-GAN (0.175), indicating the smallest perceptual difference between the generated images and the real samples. The average FID value is 8.3, superior to the other models. The CLIP score reaches 0.842, reflecting the strongest ability of style semantic alignment.

To further verify the performance of the proposed model (Model 1), it is compared with the cartoonization model based on Transformer (Model 2), the cartoon style conversion model based on CartoonGANv2 (Model 3), the cartoon style conversion model based on UGATIT (Model 4), and the cartoon style conversion model based on StyleGAN-XL (Model 5). To verify that the model maintains stable performance on real video sequences, the study selects real surveillance video clips as test data, covering three complex scenarios: day-night alternation, rain and fog weather, and dynamic occlusion. The study accelerates these video sequences to obtain three test videos, each with a duration of 30 seconds and a resolution of 1080p, and uniformly sampled them at 25 fps. These data are input into the model for style conversion, and the inter-frame consistency and cartoon style coherence are compared. In addition, the study quantitatively evaluates the cartoon style similarity, contour retention degree, and texture simplification degree of the cartoonized results of the generated animation scenes. The inter-frame consistency index is obtained by calculating the average structural similarity value between adjacent frames, the cartoon style coherence is measured by the mean cosine similarity value of the style feature vectors, and the texture simplification degree is selected as the edge gradient histogram entropy value. The experimental results are shown in Table 4.

**Table 4:** The performance of different models on real video sequences.

Model	Inter-frame consistency	Cartoon style coherence	Cartoon style similarity	Contour retention degree	Texture simplification degree(bit)
Model 1	0.93 ± 0.02^*#&@^	0.95 ± 0.01^*#&@^	0.94 ± 0.01^*#&@^	0.83 ± 0.05^*#&@^	3.65 ± 0.54^*#&@^
Model 2	0.88 ± 0.03	0.89 ± 0.03	0.88 ± 0.03	0.70 ± 0.06	4.89 ± 0.68
Model 3	0.90 ± 0.02	0.92 ± 0.02	0.91 ± 0.01	0.75 ± 0.05	4.50 ± 0.70
Model 4	0.89 ± 0.03	0.91 ± 0.02	0.90 ± 0.02	0.73 ± 0.07	4.68 ± 0.59
Model 5	0.84 ± 0.04	0.86 ± 0.03	0.85 ± 0.04	0.68 ± 0.06	5.23 ± 0.62

In Table 4, * indicates that there is a significant difference between the results of Model 1 and Model 2, with *p* < 0.05. # indicates a significant difference between Model 1 and Model 3, with *p* < 0.05. & indicates a significant difference between Model 1 and Model 4, with *p* < 0.05. @ indicates a significant difference between Model 1 and Model 5, with *p* < 0.05.

In Table 4, the inter-frame consistency, cartoon style coherence, and cartoon style similarity of Model 1 all exceed 0.93, significantly outperforming the other models. Its contour retention degree reaches 0.83, and the texture simplification degree is the lowest, at 3.65 bits. This indicates that a moderate style abstraction has been achieved while maintaining the real structural details. The simplification degree is characterized by the entropy value of the edge gradient histogram, with a lower value indicating simpler and clearer lines and textures, and higher stylization. Model 1 performs the most balancedly in all indicators, especially achieving the optimal trade-off between dynamic coherence and structural fidelity.

Human preferences and subjective opinions often play a more decisive role in evaluating the cartoonization of animation scenes. The assessment results can further demonstrate the advantages of the model in practical applications. Therefore, the research organization invites 20 animators and 15 ordinary users to participate in a double-blind test, requiring them to rank the five sets of results according to their preferences. Additionally, all participants are not informed of the corresponding model numbers and only rate independently based on visual perception and narrative fluency. The comparison indicators include preference ratio, perception score, and fluency score. The preference ratio indicator refers to the proportion of the samples ranked in the top two, while the perception score and fluency score are measured using a 5-point Likert scale. The results are shown in Table 5.

**Table 5:** Comparison of user preference and subjective evaluation results for each model.

Model	Preference ratio (%)	Perception score	Fluency score
Model 1	65.43	4.81	4.70
Model 2	32.06	3.79	3.63
Model 3	42.11	4.22	4.15
Model 4	37.20	4.00	3.95
Model 5	28.77	3.50	3.48

In Table 5, Model 1 has a preference ratio of 65.43%, which is far ahead of the others. Both the perception score and fluency score exceed 4.7 points, significantly higher than those of the other models. Compared with the other four models, Model 1 demonstrates a decisive advantage in the subjective experience dimension. Especially among the animation artists, the preference ratio is 78.2%. Among ordinary users, it is 56.3%.

The proposed model consists of several modules including the multi-style mapping module, RetNet, the Euclidean distance decreasing attention mechanism, and TOPS-GC. To evaluate the performance of each module in the overall model, the study designed ablation experiments to assess each module. During the experiment, the study removes each module one by one and retrains the complete model to quantify its contribution to the final performance. The comparison methods include: removing only the multi-style mapping module while keeping the rest of the structure (ablation 1), removing only the RetNet module (ablation 2), removing only the Euclidean distance decreasing attention mechanism (ablation 3), removing only the TPS-GG module (ablation 4), removing all the modules (baseline model ablation 5), and the Complete model. The Complete model is a complete model without any ablation components and is used as the performance benchmark. The comparison indicators include PSNR, SSIM, and FID. The comparison results are shown in Table 6.

**Table 6:** Results of the ablation experiment.

Project	PSNR	SSIM	FID
Ablation 1	32.41	0.892	24.67
Ablation 2	29.85	0.863	31.20
Ablation 3	31.07	0.879	27.34
Ablation 4	30.52	0.871	29.53
Ablation 5	27.64	0.842	35.89
Complete model	35.26	0.914	18.32

In Table 6, the complete model outperforms variant models in all indicators, with an increase of 2.85 points in PSNR, an increase of 0.022 in SSIM, and a decrease of 7.57 in FID. This confirms that the collaborative effect of each module was significantly better than the contribution of a single component. In particular, ablation 2 shows a decrease of 5.41 points in PSNR, a decrease of 0.051 in SSIM, and an increase of 12.88 in FID, making it the most severely performing ablation group among all ablations. From the above content, each module makes an undeniable contribution to the model performance, but their mechanism of action and the dimensions of influence are all different.

To test the computational complexity of the proposed method, the study evaluates the model efficiency using two dimensions: FLOPs and inference delay. The selected comparison baseline model is StyleGAN2. On the NVIDIA A100 GPU, the single-image inference time of the complete IGRAC model is 47.3 ms, with FLOPs at 18.6 G. Compared to the 62.8 ms and 24.1 G of the baseline model, the inference speed increases by 24.7%, while FLOPs decreases by 22.8%, indicating that the model significantly improves computational efficiency while maintaining high performance.

## 4. Discussion and interpretation

This study constructs an IGRAC generation model that integrates improved GAN and RetNet to address the local detail loss, inconsistent style, and structural blurring in the cartoonization generation of animated image scenes. The model has been systematically tested on two public datasets, ADE20K and Cityscapes, as well as multiple simulation environments. The proposed IGRAC performs superior in multiple key indicators, and the complete model shows significant improvements in PSNR, SSIM, and FID indicators compared to the baseline model. The ablation test further validates the key role of multi-style mapping, multi-head discriminator, and TPS-GG module in improving generation quality and structural consistency. In simulation environment testing, IGRAC maintains the highest PSNR value, processing efficiency, and lowest memory usage in four different scenarios, indicating that the model has strong generalization ability and stability. From relevant studies, Chuanyu P et al. proposed a cartoon style generation model based on semantic alignment and attention distillation, which enhances CR ability by introducing an explicit semantic constraint module. The design concept of detail preservation in this study is similar to multi-style mapping and content loss function in the research [27]. Guo M et al. developed a multi-modal cartoonization framework based on a diffusion model, which supports text guided style control. Their exploration in multi-style adaptation are in line with the design goals of the multi-style mapping module [28]. In addition, H. W. Lee et al. introduced stream models into image cartoonization tasks and achieved high fidelity texture generation through reversible transformations. The attention paid to the authenticity of generated textures and the joint optimization strategy of adversarial loss and edge preservation loss in this study are conceptually consistent with each other [29]. In current research, there are generally several bottlenecks such as weak style generalization ability, poor cross-domain transfer effect, and insufficient real-time performance [30]. In comparison, this study innovatively introduces RetNet’s sequence modeling capability into the field of image cartoonization. It enhances global structural consistency through its long-range dependency capture feature, and effectively suppresses the interference of distant irrelevant features by combining the self-attention mechanism of Euclidean distance decay. Meanwhile, the TPS-GG module explicitly constrains the smoothness and rationality of the geometric structure of the image. The IGRAC model achieves triple optimization of local texture details, diverse styles, and global structure preservation by fusing GAN and RetNet, demonstrating more comprehensive performance advantages in complex scenes. However, the proposed model mainly focus on the cartoonization transformation of static images, and there are still shortcomings in considering the temporal consistency of video sequences. Subsequent research can introduce optical flow guidance or temporal attention mechanisms to enhance inter frame stability and dynamic rendering effects in animated video scenes.

## 5. Summary

The current methods for generating cartoon-style animation image scenes have limitations in balancing high quality and low resource consumption. A new style generation model has been proposed. This model introduces RetNet to the image cartoonization task, enhancing global structural consistency through its long-range dependency capture mechanism, and effectively suppressing irrelevant distant feature interference by combining the self-attention mechanism with Euclidean distance decay. The TPS-GG module is used to constrain the smoothness and rationality of the image geometric structure. During the experimental process, when the content loss weight is 0.6 and the self-attention decay coefficient is 0.5, the IGRAC model has the fastest convergence speed and the best generation effect in the model performance test. On the ADE20K dataset, the PSNR reaches 24.8 dB, the SSIM is 0.89, and the FID drops to 35.2. All indicators are superior to the comparison model. In the simulation test, the IGRAC model shows stable adaptability in four typical scenarios, with the PSNR reaching up to 29.2 dB, the IPS up to 8.0, and the processing time as low as 5.7 seconds, with the memory usage controlled within 3.9GB. In conclusion, the IGRAC model generates high-quality cartoon-like styles in complex environments. During the training process, this model performs stably and excellently. In the testing phase, it maintains excellent generalization performance and stability in various environments. The key finding of the research is that by combining the long-range dependency modeling ability of RetNet with the local texture generation advantage of improved GAN, it is possible to effectively balance the style transfer and content preservation during the cartoonization process, thereby achieving better generation results in complex scenarios. Moreover, the IGRAC model has achieved a good balance between image quality, processing efficiency, and resource consumption, providing a feasible solution for deployment in practical applications. Even under extreme deformation, changes in lighting, or rare styles, the IGRAC model still faces detail distortion and style drift. Especially in scenes with strong shadow occlusion or surreal color schemes, the cartoonized results occasionally exhibit edge jaggedness and color discontinuities. Therefore, in the future, a multi-scale feature self-calibration mechanism and learnable color mapping constraints can be introduced, combined with a user feedback fine-tuning module, to enhance robustness and controllability under extreme conditions.
